# ALDH1A1-dopaminergic gene co-expression in human substantia nigra: meta-analysis of disease-associated correlation changes across seven independent Parkinson’s disease datasets

**DOI:** 10.3389/fnagi.2026.1806505

**Published:** 2026-05-19

**Authors:** Drake H. Harbert

**Affiliations:** Inner Architecture LLC, Canton, OH, United States

**Keywords:** ALDH1A1, alpha-synuclein, cell type enrichment, dopamine, gene expression, meta-analysis, neurodegeneration, Parkinson’s disease

## Abstract

**Background:**

Parkinson’s disease (PD) involves progressive dopaminergic neuron loss in the substantia nigra (SN). Aldehyde dehydrogenase 1A1 (ALDH1A1), the rate-limiting enzyme in retinoic acid biosynthesis, is enriched in vulnerable dopaminergic neuron subpopulations and is consistently downregulated in PD. However, the relationship between ALDH1A1 expression and broader dopaminergic pathway gene co-expression has not been systematically characterized across multiple independent datasets.

**Methods:**

Gene expression correlations were analyzed across seven independent human SN microarray datasets (*n* = 156; 70 controls, 86 PD) from the Gene Expression Omnibus. Simple arithmetic means across datasets are reported as the primary summary statistic; random-effects meta-analysis with DerSimonian-Laird estimation was applied to Fisher’s z-transformed correlation coefficients to generate pooled estimates with heterogeneity statistics. Marker gene-based enrichment scoring using published cell type markers from single-nucleus RNA-seq profiling of human substantia nigra—with all target genes excluded from signatures—was performed across six analyzable datasets. Selectivity of ALDH1A1 correlation attenuation was assessed using permutation testing (*n* = 5,000) as the primary statistical test, with parametric tests reported as supplementary.

**Results:**

In controls, ALDH1A1 showed strong co-expression with dopaminergic genes (mean r = 0.92–0.93 for TH, DDC, and SLC18A2). In PD, these correlations were attenuated (mean Δr = −0.336 for ALDH1A1-dopamine pairs). Dopamine-dopamine correlations showed less attenuation (mean Δr = −0.143). Marker gene-based enrichment scoring confirmed significant depletion of ALDH1A1-positive vulnerable dopaminergic neurons in 4 of 6 datasets. After adjusting for estimated cell type enrichment, the selectivity of ALDH1A1 attenuation was preserved (adjusted selectivity: −0.210, increased from raw selectivity of −0.190; raw permutation *p* = 0.0052).

**Conclusion:**

ALDH1A1 co-expression with dopaminergic pathway genes is attenuated in PD substantia nigra across all seven datasets examined. This attenuation is selective for ALDH1A1-containing pairs, and this selectivity persists after adjusting for cell type enrichment changes. While correlational, these findings are consistent with a role for retinoic acid pathway disruption in PD pathophysiology and warrant mechanistic investigation.

## 1 Introduction

Parkinson’s disease (PD) is the second most common neurodegenerative disorder, affecting approximately 1%–2% of individuals over age 65 worldwide (Poewe et al., 2017). The cardinal motor features—bradykinesia, rigidity, resting tremor, and postural instability—result primarily from progressive loss of dopaminergic neurons in the substantia nigra pars compacta (SNpc). By the time of clinical diagnosis, an estimated 50%–80% of SNpc dopaminergic neurons have been lost (Burke and O’Malley, 2013), underscoring the need for earlier detection and intervention.

Retinoic acid (RA), the bioactive metabolite of vitamin A, plays critical roles in nervous system development and adult neuronal maintenance. During embryogenesis, RA signaling is essential for patterning of the ventral midbrain and specification of dopaminergic progenitors (Maden, 2007; Wallén et al., 1999). In the adult brain, RA continues to participate in neuronal survival, synaptic plasticity, and gene regulation (Goodman et al., 2012). The therapeutic potential of targeting the RA pathway in PD has generated considerable interest, with all-trans retinoic acid showing neuroprotective effects in multiple PD models (Esteves et al., 2015; Jacobs et al., 2007).

Aldehyde dehydrogenase 1A1 (ALDH1A1) is the rate-limiting enzyme in retinoic acid biosynthesis, catalyzing the oxidation of retinaldehyde to retinoic acid. In the adult brain, ALDH1A1 is highly enriched in a specific subpopulation of ventral tier SNpc dopaminergic neurons (McCaffery and Dräger, 1994; Galter et al., 2006). Multiple lines of evidence establish ALDH1A1 as a marker of selective vulnerability in PD: ALDH1A1-positive dopaminergic neurons are preferentially lost (Liu et al., 2014; Kamath et al., 2022), ALDH1A1 mRNA and protein are consistently downregulated in PD substantia nigra (Grünblatt et al., 2004; Mandel et al., 2005; Galter et al., 2006), and ALDH1A1 deficiency impairs dopamine homeostasis and nigrostriatal function in animal models (Anderson et al., 2011; Sgobio et al., 2017).

Recent single-nucleus RNA sequencing studies have refined our understanding of dopaminergic neuron heterogeneity in the human substantia nigra. Kamath et al. (2022) identified a SOX6-positive/AGTR1-positive dopaminergic neuron subpopulation that selectively degenerates in PD, and this vulnerable population overlaps substantially with ALDH1A1-expressing neurons. These findings, combined with the established role of RA in neuronal maintenance, raise the question of whether ALDH1A1 is merely a passive marker of a vulnerable cell type or whether its enzymatic function—and therefore RA production—actively contributes to dopaminergic neuron maintenance.

While previous studies have examined ALDH1A1 expression levels and identified co-expressed genes in healthy dopaminergic neurons (La Manno et al., 2016), the systematic relationship between ALDH1A1 and core dopaminergic pathway genes—and how these relationships change in disease—has not been quantified across multiple independent datasets. Such cross-dataset analysis is important because individual microarray studies of postmortem substantia nigra have limited sample sizes and may be subject to batch effects, platform differences, and other technical artifacts.

The neurodegenerative process in PD has been difficult to model, and the repeated failure of neuroprotective clinical trials predicated on preclinical models has prompted reconsideration of the cellular mechanisms involved (Kalia et al., 2015; Athauda and Foltynie, 2015). Among current theoretical frameworks, the cell-autonomous degeneration model—in which endogenous neurotoxin formation within individual neuromelanin-containing dopaminergic neurons initiates a restricted, cell-by-cell degenerative process rather than a spreading inter-cellular pathology (Huenchuguala and Segura-Aguilar, 2023)—is particularly relevant for the interpretation of correlational findings across a heterogeneous dopaminergic neuron population. Under a cell-autonomous framework, bulk tissue correlation structure reflects the statistical aggregation of many independent cellular states rather than a coordinated network-level disease process, and the attenuation of ALDH1A1-dopamine co-expression we report is consistent with, though does not adjudicate between, cell-autonomous and network-level interpretations. The present analysis is correlational and does not commit to a specific mechanistic model; we engage the cell-autonomous framework here to provide theoretical context for the interpretation of our findings.

In this study, we performed a systematic meta-analysis of gene expression correlations between ALDH1A1 and dopaminergic pathway genes across seven independent human substantia nigra microarray datasets, comprising 156 samples from 70 neurologically normal controls and 86 PD patients. We compared ALDH1A1-dopamine correlation changes to dopamine-dopamine pair changes as an internal control, and performed marker gene-based enrichment scoring to assess whether observed patterns could be attributed to altered cell type composition. All analyses are correlational and observational; causal relationships cannot be established from these data.

## 2 Materials and methods

### 2.1 Literature search and dataset selection

This systematic review and meta-analysis was conducted following PRISMA guidelines (Page et al., 2021). The Gene Expression Omnibus (GEO) database was searched in September 2024 using the following strategy: (“Parkinson” OR “Parkinson’s disease”) AND (“substantia nigra”) AND (“Homo sapiens”[Organism]). Inclusion criteria were: (1) human postmortem brain tissue samples; (2) substantia nigra region specifically dissected and annotated; (3) case-control study design with both neurologically normal controls and PD patients; (4) genome-wide expression profiling using Affymetrix microarray platforms; and (5) publicly available processed expression data. Exclusion criteria were: (1) cell line or animal model studies; (2) laser-capture microdissection of specific cell populations; (3) brain regions other than substantia nigra; and (4) fewer than five samples per group. Seven datasets meeting all criteria were identified: GSE7621, GSE8397, GSE20163, GSE20164, GSE20292, GSE20333, and GSE49036 (Table 1).

**TABLE 1:** Characteristics of datasets included in the meta-analysis.

Dataset	Platform	Year	Ctrl *n*	PD *n*	Total *n*	Brain region	References
GSE7621	GPL570 (HG-U133 Plus 2.0)	2007	9	16	25	Substantia nigra	Lesnick et al.
GSE8397^1^	GPL96 (HG-U133A)	2007	15	24	39	Substantia nigra	Moran et al.
GSE20163	GPL96 (HG-U133A)	2010	9	8	17	Substantia nigra	Zheng et al.
GSE20164	GPL96 (HG-U133A)	2010	5	6	11	Substantia nigra	Zheng et al.
GSE20292	GPL96 (HG-U133A)	2010	18	11	29	Substantia nigra	Stamper et al.
GSE20333	GPL201 (HG-Focus)	2010	6	6	12	Substantia nigra	Zheng et al.
GSE49036^2^	GPL570 (HG-U133 Plus 2.0)	2013	8	15	23	Substantia nigra	Dumitriu et al.
Total			70	86	156	–	–

^1^GSE8397 profiled each sample on both HG-U133A and HG-U133B arrays. Only HG-U133A data from substantia nigra samples were retained.

^2^GSE49036: Only idiopathic PD and neurologically normal control samples retained; iLBD, PDD, and DLB samples excluded.

### 2.2 Target gene selection

Target genes were selected *a priori* based on established roles in dopamine biosynthesis, vesicular transport, synaptic reuptake, and PD pathophysiology. The primary gene of interest was ALDH1A1 (aldehyde dehydrogenase 1 family member A1; rate-limiting enzyme in retinoic acid biosynthesis). Dopaminergic pathway genes included: TH (tyrosine hydroxylase; rate-limiting enzyme in dopamine synthesis), DDC (aromatic L-amino acid decarboxylase; converts L-DOPA to dopamine), SLC18A2 (vesicular monoamine transporter 2; packages dopamine into synaptic vesicles), and SLC6A3 (dopamine transporter; mediates synaptic reuptake). SNCA (α-synuclein; aggregation of which forms Lewy bodies in PD) was included as a PD-associated gene that is co-expressed with dopaminergic genes but implicated in disease pathogenesis through distinct mechanisms.

### 2.3 Data acquisition and processing

Processed expression matrices (series matrix files) and sample annotations were downloaded from GEO using the GEOparse package in Python. Expression values were log2-transformed where not already transformed, and quantile-normalized within each dataset. Probe-to-gene mapping was performed using platform annotation files; where multiple probes mapped to the same gene, the probe with the highest mean expression across all samples was retained. Sample group assignments (control vs. PD) were extracted from sample annotations and verified against original publications. For GSE8397, which profiled each biological sample on both HG-U133A and HG-U133B companion arrays and contains samples from multiple brain regions, only HG-U133A data from substantia nigra samples were retained to avoid duplicate profiling and ensure tissue specificity. All datasets used Affymetrix platforms (GPL96, GPL97, GPL201, or GPL570), ensuring methodological consistency across the meta-analysis.

Microarray datasets were prioritized over RNA-seq for three reasons. First, the public repository of human substantia nigra case-control PD datasets is weighted toward microarray platforms (Affymetrix HG-U133 series, 2006–2013), with RNA-seq SN case-control PD bulk data remaining comparatively sparse at the time of this analysis. Selecting microarray datasets allowed the meta-analysis to include seven independent cohorts rather than two or three, substantially improving statistical precision and cross-study replication. Second, restricting to a single measurement modality avoids the need to harmonize expression-unit differences between platforms, which can introduce technical artifacts into correlation structure. Third, the Affymetrix HG-U133 platforms have well-characterized probe-to-gene mappings and stable normalization procedures, supporting reproducible cross-dataset comparisons. Extension of this analysis to emerging RNA-seq SN datasets is identified as a future direction (see section “4.6 Future directions”).

### 2.4 Correlation analysis

Within each dataset, Pearson product-moment correlation coefficients were calculated between all pairs of target genes separately for control and PD sample groups using Python 3.10 with scipy (version 1.11.0). Correlation change (Δr) was calculated as the difference between PD and control correlations (Δr = r_PD−r_control), such that negative values indicate attenuation in disease. No minimum sample size threshold was imposed beyond the dataset inclusion criterion of *n* ≥ 5 per group, as the meta-analytic framework accounts for precision differences through inverse-variance weighting.

### 2.5 Meta-analysis

Meta-analysis of correlation coefficients followed established methods (Borenstein et al., 2009). Individual Pearson correlations were transformed using Fisher’s z-transformation: z = 0.5 × ln[(1 + r)/(1−r)], with standard error SE = 1/√(n−3). Random-effects meta-analysis was performed using the DerSimonian-Laird estimator (DerSimonian and Laird, 1986) to pool z-transformed correlations across datasets, with pooled z subsequently back-transformed to correlation coefficients. Heterogeneity was assessed using Cochran’s Q statistic and the I^2^ index (Higgins et al., 2003), where I^2^ < 25% indicates low heterogeneity, 25%–75% moderate, and >75% high. Simple arithmetic means across datasets are additionally reported as a more conservative and interpretable summary statistic. Statistical comparison of control versus PD pooled correlations was performed using the z-test for comparing two independent correlations. Meta-analysis was implemented in Python using custom code following the formulas in Borenstein et al. (2009); complete analysis code is publicly available (see Data Availability Statement).

### 2.6 Selectivity analysis

To quantify selectivity of correlation changes, gene pairs were classified into three categories: ALDH1A1-dopamine pairs (ALDH1A1 paired with TH, DDC, SLC18A2, or SLC6A3; *n* = 4 pairs), dopamine-dopamine pairs (all pairwise combinations among TH, DDC, SLC18A2, and SLC6A3; *n* = 6 pairs), and SNCA-containing pairs (SNCA paired with any dopaminergic gene; analyzed separately due to its distinct biological role). Because gene pairs sharing a common node (e.g., ALDH1A1-TH and ALDH1A1-DDC) violate the independence assumption required by parametric comparison tests, permutation testing was designated as the primary statistical test for selectivity (see section “2.7 Cell type enrichment analysis”), with parametric tests (Welch’s *t*-test, Mann-Whitney U test) reported as supplementary. Effect size was quantified using Cohen’s *d*.

### 2.7 Cell type enrichment analysis

To address the potential confound of altered cell type composition in PD substantia nigra, marker gene-based enrichment scoring was performed using cell type-specific marker genes derived from published single-nucleus RNA-seq profiling of human substantia nigra (Kamath et al., 2022). Cell type signatures were defined for eight populations: ALDH1A1-positive vulnerable dopaminergic neurons (SOX6 + /AGTR1 + subtype; 20 marker genes), ALDH1A1-negative resistant dopaminergic neurons (CALB1 + subtype; 15 markers), GABAergic neurons (13 markers), glutamatergic neurons (9 markers), astrocytes (13 markers), microglia (13 markers), oligodendrocytes (12 markers), and endothelial cells (10 markers). Critically, all six target genes (ALDH1A1, TH, DDC, SLC18A2, SLC6A3, SNCA) were excluded from all cell type signatures to prevent circularity between the enrichment scoring and correlation analyses (see Supplementary Table 4 for complete marker gene lists).

Cell type enrichment scores were computed as the mean z-scored expression of marker genes per sample, analogous to the approach used by xCell (Aran et al., 2017). Formal proportion estimates were additionally obtained via non-negative least squares (NNLS) regression, which is mathematically equivalent to the core algorithm of CIBERSORTx (Newman et al., 2019). This approach provides approximate rather than precise cell type proportion estimates; it is described throughout as “marker gene-based enrichment scoring” to distinguish it from full reference-based deconvolution using complete single-cell transcriptome profiles. Partial Pearson correlations were then computed controlling for estimated ALDH1A1-positive dopaminergic neuron enrichment to assess whether the selectivity of ALDH1A1 correlation attenuation persisted after accounting for cell type enrichment changes. GSE7621 was excluded from the enrichment analysis due to insufficient marker gene coverage on this platform configuration (see section “3.1 Dataset characteristics”), yielding six analyzable datasets.

Selectivity of ALDH1A1 correlation attenuation was tested using a permutation-based approach (*n* = 5,000 permutations) that shuffled disease labels at the sample level within each dataset. This procedure preserves the dependency structure among gene pairs sharing common nodes and provides a non-parametric *p*-value that does not assume independence of gene pair observations, directly addressing the concern that parametric tests applied to gene pair Δr values may violate independence assumptions.

### 2.8 Negative control gene pairs

Housekeeping gene pairs (GAPDH-ACTB, RPL13A-RPS18, HPRT1-B2M, UBC-PPIA) were included as negative controls to provide context for the magnitude of observed correlation changes. These pairs were selected because they are commonly used normalization references and their co-expression is expected to be relatively stable across conditions. Comparison of dopaminergic and housekeeping gene pair correlation changes provides an empirical benchmark for the degree of transcriptomic instability in PD tissue.

### 2.9 Sensitivity analysis

Leave-one-out sensitivity analysis was performed to assess whether any single dataset disproportionately influenced meta-analysis results. For each of the seven datasets, meta-analysis was recalculated with that dataset excluded. Additionally, selectivity was evaluated across multiple dataset subsets, including restriction to the four datasets showing significant dopaminergic neuron depletion and exclusion of datasets with potential quality concerns (GSE20292, which exhibited extreme housekeeping gene correlation instability, and GSE20333, which showed non-significant dopaminergic neuron depletion). Selectivity was additionally computed excluding SLC6A3 from the ALDH1A1-dopamine category to assess whether the heterogeneous behavior of this gene influenced the primary findings.

### 2.10 Statistical thresholds and software

Correlations were classified by magnitude as strong (|r| ≥ 0.7), moderate (0.5 ≤ |r| < 0.7), or weak (|r| < 0.5). Two-sided *p*-values < 0.05 were considered statistically significant. Formal assessment of publication bias using funnel plots or Egger’s test was not performed due to the limited number of included studies (*n* = 7); with fewer than 10 studies, such tests have low statistical power and are not recommended (Sterne et al., 2011). All analyses were performed using Python 3.10 (scipy 1.11.0, pandas 2.0.0, numpy 1.24.0, matplotlib 3.7.0). Cell type enrichment analysis was performed using Python with scipy.optimize (NNLS implementation). Complete analysis code is publicly available (see Data Availability Statement).

### 2.11 Ethics statement

This study involved secondary analysis of publicly available, de-identified gene expression data from the Gene Expression Omnibus. All original studies obtained appropriate institutional ethics approvals and informed consent. No additional ethics approval was required for this secondary analysis.

## 3 Results

### 3.1 Dataset characteristics

Seven datasets meeting inclusion criteria were identified from 47 initial records (Supplementary Figure 1), comprising 156 substantia nigra samples: 70 from neurologically normal controls and 86 from PD patients (Table 1). Individual dataset sample sizes ranged from 11 (GSE20164) to 39 (GSE8397, substantia nigra A-chip subset). All datasets used Affymetrix microarray platforms spanning publication years 2007 to 2013. Quality assessment of included datasets is reported in Supplementary Table 1. All six target genes were detected in every dataset. Correlation analyses were performed across all seven datasets. Cell type enrichment analysis was performed across six datasets (GSE7621) was excluded from the cell type enrichment analysis because probe-to-gene mapping for the Kamath et al. (2022) marker gene set yielded insufficient marker coverage on this platform configuration, resulting in unreliable enrichment scores. GSE7621 is retained in the primary correlation meta-analysis, which requires only the six target genes). Selectivity analyses are reported for both the full seven-dataset correlation sample and the six-dataset subset used for cell type adjustment, with consistent results.

### 3.2 ALDH1A1 co-expression with dopaminergic genes in control substantia nigra

**FIGURE 1:**
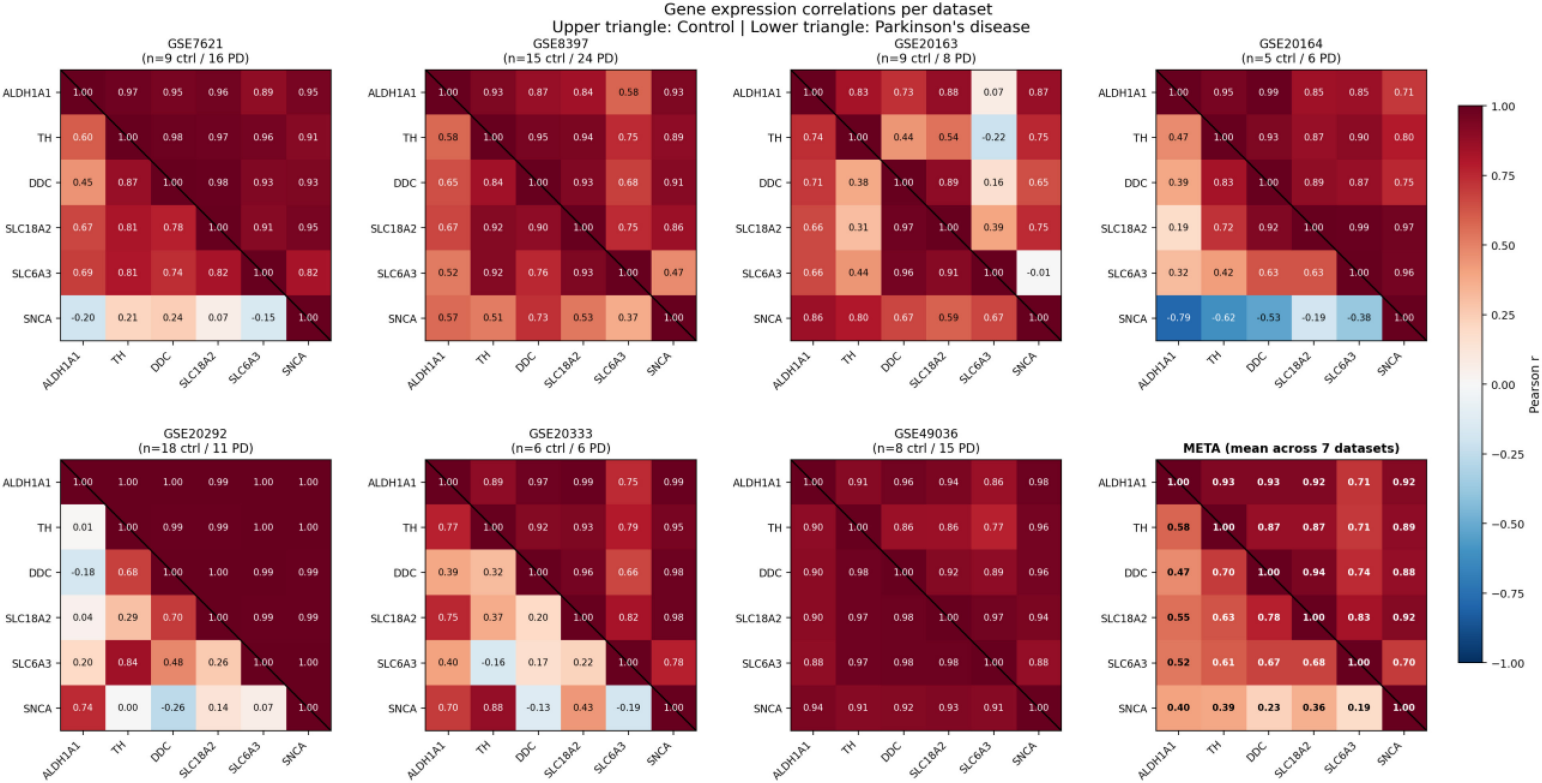
Gene expression correlations per dataset. Eight-panel grid showing Pearson correlation coefficients among six target genes (ALDH1A1, TH, DDC, SLC18A2, SLC6A3, SNCA) across the seven individual datasets and the meta-analytic mean (META). Within each panel, the upper triangle represents control samples and the lower triangle represents Parkinson’s disease samples, separated by a diagonal line. Color scale: blue (negative) to red (positive) Pearson correlation. Cell annotations show correlation values to two decimal places. Panel titles report control and PD sample counts. A shared colorbar appears at right.

In neurologically normal control samples, ALDH1A1 expression showed consistently strong positive correlations with all examined dopaminergic pathway genes across datasets (Table 2). The mean correlation coefficients across seven datasets were: ALDH1A1-TH (mean *r* = 0.927), ALDH1A1-DDC (mean *r* = 0.923), ALDH1A1-SLC18A2 (mean *r* = 0.923), and ALDH1A1-SLC6A3 (mean *r* = 0.715). ALDH1A1-SNCA also showed strong co-expression (mean *r* = 0.867). Pooled random-effects estimates with 95% confidence intervals and heterogeneity statistics are provided in Table 2B. Dopaminergic pathway genes demonstrated strong mutual correlations: DDC-SLC18A2 (mean *r* = 0.934), SLC18A2-TH (mean *r* = 0.883), DDC-TH (mean *r* = 0.869), and SLC18A2-SLC6A3 (mean *r* = 0.817). Per-dataset correlation values for all gene pairs in control and PD samples are reported in Supplementary Table 2. These high inter-correlations among dopaminergic genes are consistent with their co-expression within the same cell type and shared transcriptional regulation. Per-dataset correlation matrices are shown in Figure 1.

**TABLE 2A:** Mean correlation coefficients across seven datasets.

Gene pair	Category	Mean *r* ctrl	Mean r PD	Mean Δr	Attenuation
ALDH1A1-TH	ALDH1A1-DA	0.927	0.582	−0.345	Strong
ALDH1A1-DDC	ALDH1A1-DA	0.923	0.477	−0.446	Strong
ALDH1A1-SLC18A2	ALDH1A1-DA	0.923	0.560	−0.363	Strong
ALDH1A1-SLC6A3	ALDH1A1-DA	0.715	0.525	−0.189	Moderate
DDC-SLC18A2	DA-DA	0.934	0.769	−0.165	Moderate
TH-DDC	DA-DA	0.869	0.708	−0.161	Moderate
TH-SLC18A2	DA-DA	0.883	0.638	−0.245	Moderate
SLC18A2-SLC6A3	DA-DA	0.817	0.681	−0.136	Moderate
DDC-SLC6A3	DA-DA	0.731	0.679	−0.052	Mild
SLC6A3-TH	DA-DA	0.708	0.610	−0.098	Mild
DDC-SNCA	SNCA	0.851	0.178	−0.672	Strong
SLC18A2-SNCA	SNCA	0.917	0.351	−0.566	Strong
SLC6A3-SNCA	SNCA	0.730	0.199	−0.531	Strong
TH-SNCA	SNCA	0.853	0.361	−0.492	Strong
ALDH1A1-SNCA	SNCA	0.867	0.391	−0.476	Strong
ALDH1A1-DA mean (4 pairs)	–	–	–	−0.336	–
DA-DA mean (6 pairs)	–	–	–	−0.143	–
SNCA mean (5 pairs)	–	–	–	−0.548	–

Values represent arithmetic means of Pearson correlation coefficients across seven independent substantia nigra microarray datasets (*n* = 156 total; 70 controls, 86 PD). Δr = PD–Control.

**TABLE 2B:** Pooled random-effects meta-analysis estimates with 95% confidence intervals and heterogeneity statistics for all gene pairs.

Gene pair	Condition	Pooled r	95% CI	I^2^ (%)	Q	Q p	Category
ALDH1A1-TH	Ctrl	0.956	[0.846, 0.988]	79.6	29.48	<0.001	ALDH1A1-DA
ALDH1A1-TH	PD	0.643	[0.386, 0.808]	46.4	11.19	0.083	ALDH1A1-DA
ALDH1A1-DDC	Ctrl	0.963	[0.867, 0.990]	79.7	29.53	<0.001	ALDH1A1-DA
ALDH1A1-DDC	PD	0.574	[0.205, 0.800]	65.4	17.35	0.008	ALDH1A1-DA
ALDH1A1-SLC18A2	Ctrl	0.955	[0.857, 0.987]	76.1	25.08	<0.001	ALDH1A1-DA
ALDH1A1-SLC18A2	PD	0.666	[0.371, 0.839]	60.1	15.04	0.020	ALDH1A1-DA
ALDH1A1-SLC6A3	Ctrl	0.871	[0.232, 0.985]	92.9	84.59	<0.001	ALDH1A1-DA
ALDH1A1-SLC6A3	PD	0.619	[0.387, 0.777]	33.0	8.95	0.176	ALDH1A1-DA
TH-DDC	Ctrl	0.933	[0.811, 0.977]	70.8	20.55	0.002	DA-DA
TH-DDC	PD	0.821	[0.653, 0.912]	52.5	12.63	0.049	DA-DA
TH-SLC18A2	Ctrl	0.936	[0.838, 0.976]	62.9	16.17	0.013	DA-DA
TH-SLC18A2	PD	0.785	[0.497, 0.917]	74.1	23.19	0.001	DA-DA
TH-SLC6A3	Ctrl	0.883	[0.352, 0.984]	91.7	72.51	<0.001	DA-DA
TH-SLC6A3	PD	0.805	[0.540, 0.925]	73.6	22.69	0.001	DA-DA
DDC-SLC18A2	Ctrl	0.970	[0.824, 0.995]	89.9	59.47	<0.001	DA-DA
DDC-SLC18A2	PD	0.881	[0.729, 0.950]	66.8	18.09	0.006	DA-DA
DDC-SLC6A3	Ctrl	0.850	[0.450, 0.966]	85.3	40.90	<0.001	DA-DA
DDC-SLC6A3	PD	0.798	[0.592, 0.906]	59.6	14.85	0.021	DA-DA
SLC18A2-SLC6A3	Ctrl	0.907	[0.662, 0.977]	82.7	34.63	<0.001	DA-DA
SLC18A2-SLC6A3	PD	0.831	[0.592, 0.936]	73.9	22.95	0.001	DA-DA
ALDH1A1-SNCA	Ctrl	0.959	[0.833, 0.990]	83.5	36.42	<0.001	SNCA
ALDH1A1-SNCA	PD	0.584	[−0.050, 0.883]	87.1	46.50	<0.001	SNCA
TH-SNCA	Ctrl	0.934	[0.747, 0.984]	83.2	35.81	<0.001	SNCA
TH-SNCA	PD	0.516	[0.026, 0.806]	77.1	26.16	<0.001	SNCA
DDC-SNCA	Ctrl	0.946	[0.852, 0.981]	67.6	18.52	0.005	SNCA
DDC-SNCA	PD	0.348	[−0.201, 0.731]	78.9	28.46	<0.001	SNCA
SLC18A2-SNCA	Ctrl	0.953	[0.882, 0.981]	60.3	15.11	0.019	SNCA
SLC18A2-SNCA	PD	0.488	[−0.005, 0.791]	76.6	25.61	<0.001	SNCA
SLC6A3-SNCA	Ctrl	0.882	[0.250, 0.987]	93.3	89.15	<0.001	SNCA
SLC6A3-SNCA	PD	0.338	[−0.145, 0.691]	72.5	21.79	0.001	SNCA

Pooled estimates computed via DerSimonian-Laird random-effects meta-analysis of Fisher’s z-transformed correlations, back-transformed to Pearson r. I^2^: percentage of variability due to heterogeneity rather than chance (<25% low, 25%–75% moderate, >75% high). Q: Cochran’s Q heterogeneity statistic. k = 7 datasets for all pairs. Control I^2^ values are moderate-to-high for most pairs, reflecting genuine biological variability across datasets. PD I^2^ values for ALDH1A1-containing pairs are generally lower than controls, consistent with cross-dataset consistency of attenuation (convergence toward similar PD correlation values). Several SNCA-PD confidence intervals include zero, reflecting high between-dataset variability in SNCA correlation changes.

**FIGURE 2:**
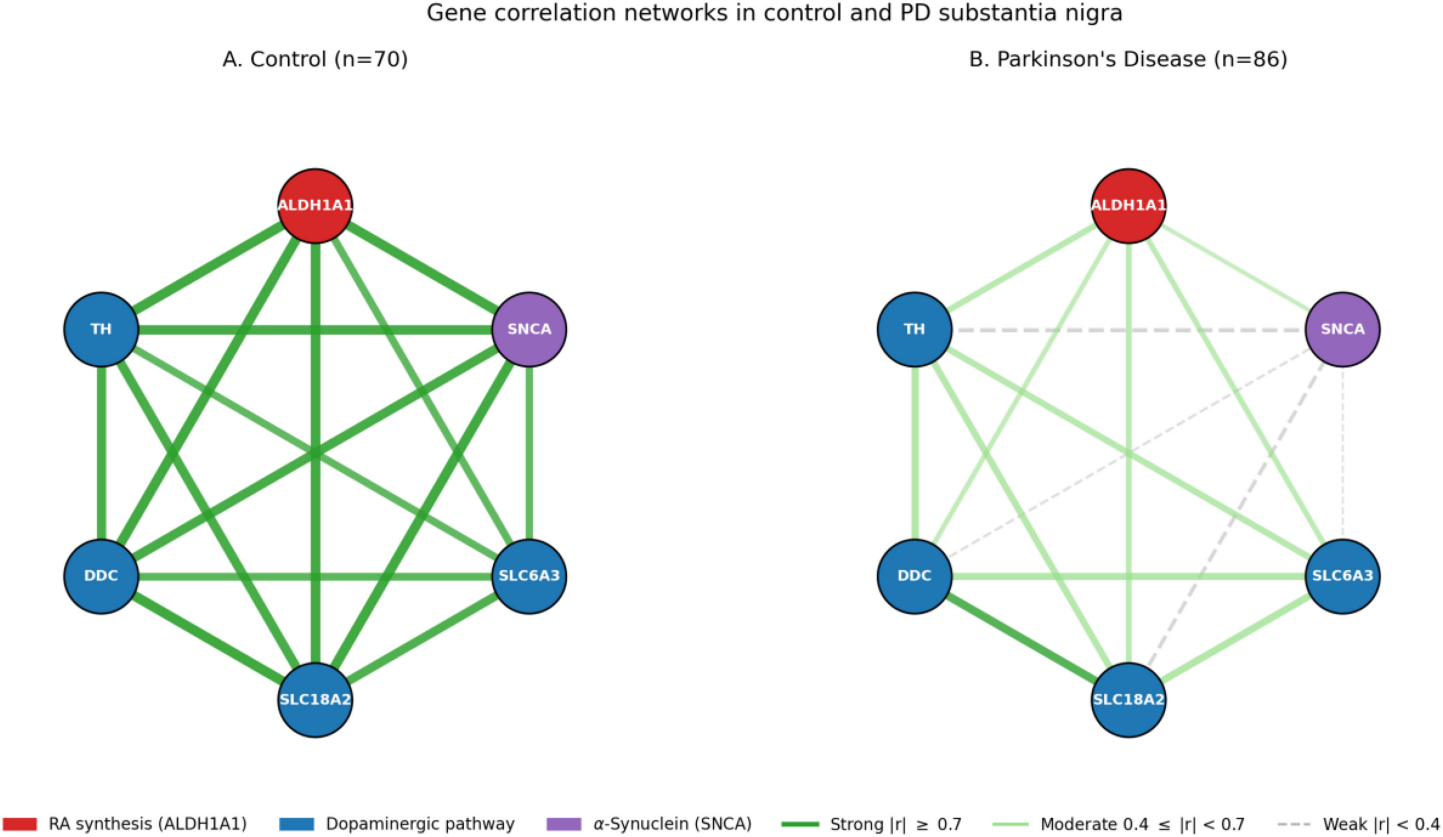
Gene correlation networks in control **(A)** and Parkinson’s disease **(B)** substantia nigra. Each node represents a target gene (ALDH1A1 in red as the retinoic acid synthesis marker; dopaminergic pathway genes TH, DDC, SLC18A2, SLC6A3 in blue; SNCA in purple). Edges are drawn for all pairwise correlations; edge width scales with |r| and edges are stratified into three classes: strong (|r| ≥ 0.7; saturated green solid), moderate (0.4 ≤ |r| < 0.7; pale green solid), and weak (|r| < 0.4; gray dashed).

The moderate-to-high I^2^ values observed for control correlations (Table 2B; range: 60%–93%) reflect genuine biological and technical variability across datasets arising from differences in sample sizes, microarray platforms, tissue dissection precision, and cohort demographics. Notably, PD I^2^ values for ALDH1A1-containing pairs are generally lower than their control counterparts, consistent with disease driving convergence toward a common attenuated state across diverse cohorts. The consistency of attenuation direction across all seven datasets provides qualitative support independent of the precise magnitude of any individual estimate. Network visualizations of control and PD correlation structure are shown in Figure 2.

### 3.3 ALDH1A1 correlations are attenuated in Parkinson’s disease

In PD substantia nigra samples, correlations between ALDH1A1 and dopaminergic genes showed consistent attenuation across all seven datasets (Table 2): ALDH1A1-TH (mean *r* = 0.582, Δr = −0.345), ALDH1A1-DDC (mean *r* = 0.477, Δr = −0.446), ALDH1A1-SLC18A2 (mean *r* = 0.560, Δr = −0.363), and ALDH1A1-SLC6A3 (mean *r* = 0.525, Δr = −0.189). ALDH1A1-SNCA showed the largest attenuation (mean *r* = 0.391, Δr = −0.476). The mean Δr for ALDH1A1-dopamine pairs (ALDH1A1-TH, ALDH1A1-DDC, ALDH1A1-SLC18A2, ALDH1A1-SLC6A3) was −0.336.

**FIGURE 3:**
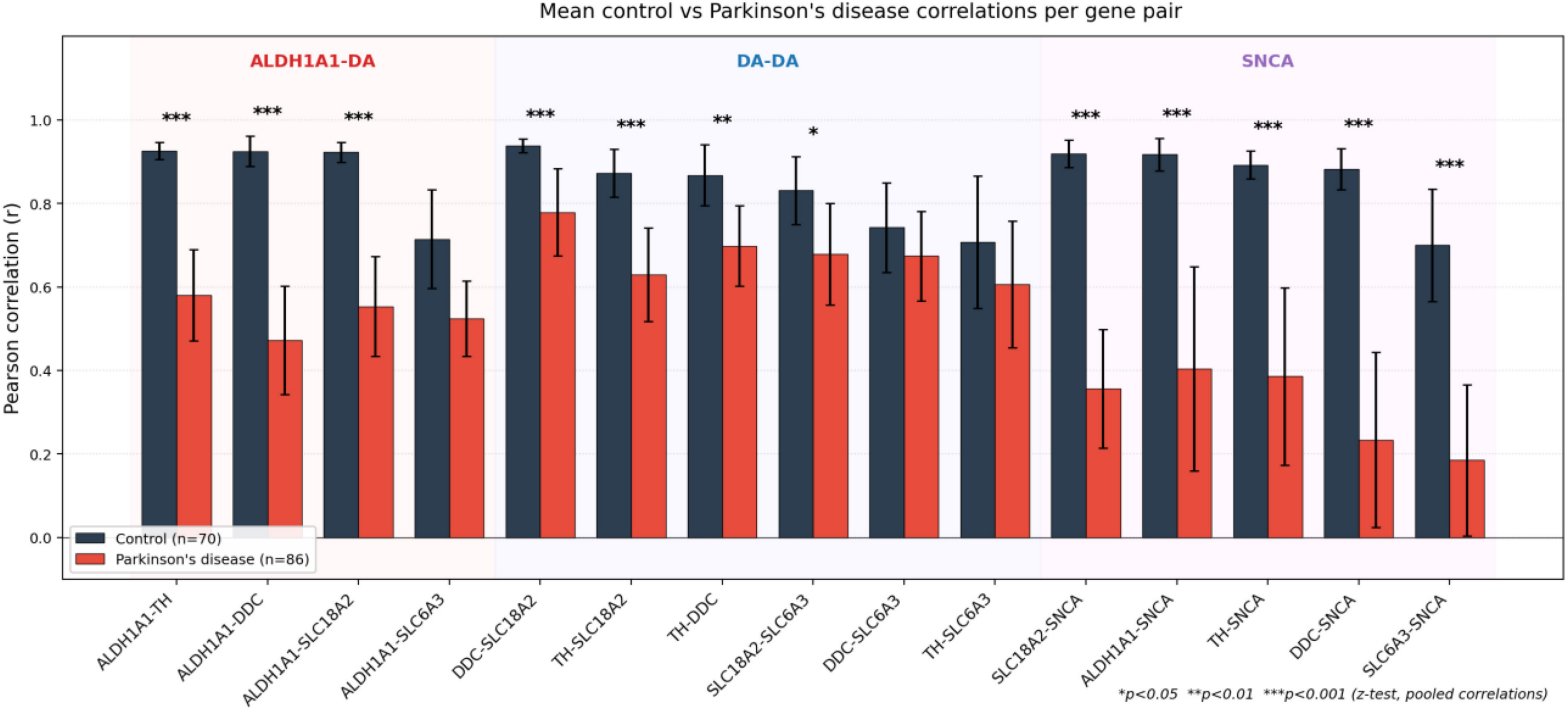
Mean control vs. Parkinson’s disease correlations per gene pair. Bars show mean Pearson r across seven datasets for control (dark gray) and PD (red) samples; error bars indicate standard error of the mean. Pairs are grouped into three category blocks (ALDH1A1-DA, DA-DA, SNCA) with background shading. Asterisks above bars indicate significance of the z-test comparing pooled control and PD correlations (*p < 0.05, **p < 0.01, ***p < 0.001).

Z-tests comparing the pooled control and PD correlation coefficients are reported as descriptive per-pair comparisons. These tests compare meta-analytic pooled correlations within a single gene pair (where the two groups, control and PD, are independent samples) and do not address the primary selectivity question (ALDH1A1-DA vs. DA-DA pair categories), because the gene pairs within each category are non-independent—sharing common nodes (e.g., ALDH1A1-TH and ALDH1A1-DDC share ALDH1A1). The primary test of the selectivity hypothesis is the sample-level permutation test in section “3.6 Selectivity of ALDH1A1 correlation attenuation,” which preserves this non-independence structure by shuffling disease labels within each dataset. With this caveat, the per-pair z-tests indicated attenuation in three of four ALDH1A1-dopamine pairs (ALDH1A1-TH: *z* = 3.45, *p* < 0.001; ALDH1A1-SLC18A2: *z* = 3.06, *p* = 0.002; ALDH1A1-DDC: *z* = 2.92, *p* = 0.004), with ALDH1A1-SLC6A3 attenuated but non-significant (*p* = 0.370; consistent with the heterogeneous SLC6A3 behavior discussed in section “4.2 Selective attenuation in Parkinson’s disease”), and none of the six dopamine-dopamine pairs reaching nominal significance (all *p* > 0.18; Supplementary Table 8). Mean control versus PD correlations per gene pair are shown in Figure 3.

### 3.4 Dopamine-dopamine correlations show less attenuation

In contrast to ALDH1A1 correlations, correlations among dopaminergic pathway genes themselves showed less attenuation in PD (Table 2). DDC-SLC18A2 (mean *r* = 0.769, Δr = −0.165), DDC-TH (mean *r* = 0.708, Δr = −0.161), SLC18A2-TH (mean *r* = 0.638, Δr = −0.245), SLC18A2-SLC6A3 (mean *r* = 0.681, Δr = −0.136), DDC-SLC6A3 (mean *r* = 0.679, Δr = −0.052), and SLC6A3-TH (mean *r* = 0.610, Δr = −0.098). The mean Δr for all six dopamine-dopamine pairs was −0.143, smaller in magnitude than the ALDH1A1 pair mean of −0.336.

### 3.5 SNCA-containing pairs show intermediate to large attenuation

SNCA-containing pairs exhibited the largest attenuation magnitudes: DDC-SNCA (Δr = −0.672), SNCA-TH (Δr = −0.492), SLC18A2-SNCA (Δr = −0.566), SLC6A3-SNCA (Δr = −0.531), and ALDH1A1-SNCA (Δr = −0.476). The mean Δr for SNCA-dopamine pairs was −0.548. The larger SNCA attenuation is consistent with α-synuclein’s central role in PD pathogenesis and may reflect a combination of cell loss and disease-specific pathological processes. Because SNCA pairs showed uniformly large attenuation across all partner genes—regardless of whether the partner was ALDH1A1 or a core dopaminergic gene—SNCA attenuation does not exhibit the selectivity pattern that is the focus of this analysis and was therefore analyzed separately from the ALDH1A1 vs. dopamine-dopamine selectivity comparison. The SNCA findings warrant dedicated investigation and may reflect disease-specific transcriptional consequences of synuclein pathology rather than cell-type-specific vulnerability.

#### 3.5.1 Triangulation against non-specific transcriptional collapse

A central question raised by these findings is whether ALDH1A1 attenuation reflects a biologically specific signal or is part of a generalized transcriptional collapse in PD substantia nigra. Three independent baselines argue against a non-specific-collapse explanation. First, dopamine-dopamine pairs (*n* = 6 pairs; mean Δr = −0.143) serve as a within-pathway comparator: these genes are themselves dopaminergic but are not paired with ALDH1A1, and their attenuation is less than half of ALDH1A1-dopamine pair attenuation (mean Δr = −0.336; ratio ≈ 2.3×). Second, housekeeping gene pairs (see section “3.8 Negative control gene pairs”; mean Δr = −0.106 across enrichment-analyzable datasets) provide an off-pathway baseline for generalized tissue-level correlation instability; ALDH1A1 attenuation exceeds this baseline by a factor of approximately 3×. Third, SNCA-containing pairs (mean Δr = −0.548) show a different pattern again: uniformly large attenuation regardless of partner, consistent with a global disease-process signal rather than a partner-specific effect. ALDH1A1 falls between these patterns—larger than dopamine-dopamine and housekeeping attenuation, but smaller and more selective than SNCA—which is not the signature of a non-specific collapse. The selectivity of ALDH1A1 attenuation against three distinct baselines (within-pathway, off-pathway, and disease-global) is consistent with a biologically specific signal rather than a global transcriptional dissolution.

### 3.6 Selectivity of ALDH1A1 correlation attenuation

The central question is whether ALDH1A1-containing pairs show greater attenuation than dopamine-dopamine pairs. Because gene pairs sharing a common node violate the independence assumption of parametric tests, the primary test of selectivity was a permutation approach (*n* = 5,000) that shuffled disease labels at the sample level within each dataset, preserving the dependency structure among gene pairs.

Using data from the six datasets included in the enrichment analysis, the mean per-dataset Δr for ALDH1A1-dopamine pairs (−0.338, *n* = 24 per-dataset observations across 6 datasets × 4 pairs) was compared to dopamine-dopamine pairs (−0.147, *n* = 36 per-dataset observations across 6 datasets × 6 pairs). The observed raw selectivity difference (ALDH1A1 minus DA-DA mean Δr) was −0.190. Permutation testing yielded *p* = 0.0052, with the observed selectivity 3.4 standard deviations from the permutation null mean, indicating that the pattern is unlikely to arise from chance. The effect size was moderate-to-large (Cohen’s *d* = −0.475). Supplementary parametric tests were consistent in direction (Welch’s *t* = −1.788, *p* = 0.080; Mann-Whitney U = 310, *p* = 0.067), though the non-significance of these tests reflects their limited statistical power with *n* = 4 vs. *n* = 6 gene pair categories and the violation of independence assumptions that motivated the permutation approach. When selectivity was computed across all seven datasets, the parametric comparison reached conventional significance (Welch’s *t* = −2.114, *p* = 0.039; Cohen’s *d* = −0.520).

The permutation test achieves greater statistical power than the parametric comparisons because it leverages within-dataset sample-level variation across all gene pairs simultaneously, rather than reducing the data to per-pair summary statistics (*n* = 4 vs. *n* = 6 categories) where parametric tests have limited power regardless of the true effect.

### 3.7 Cell type enrichment analysis

**FIGURE 4:**
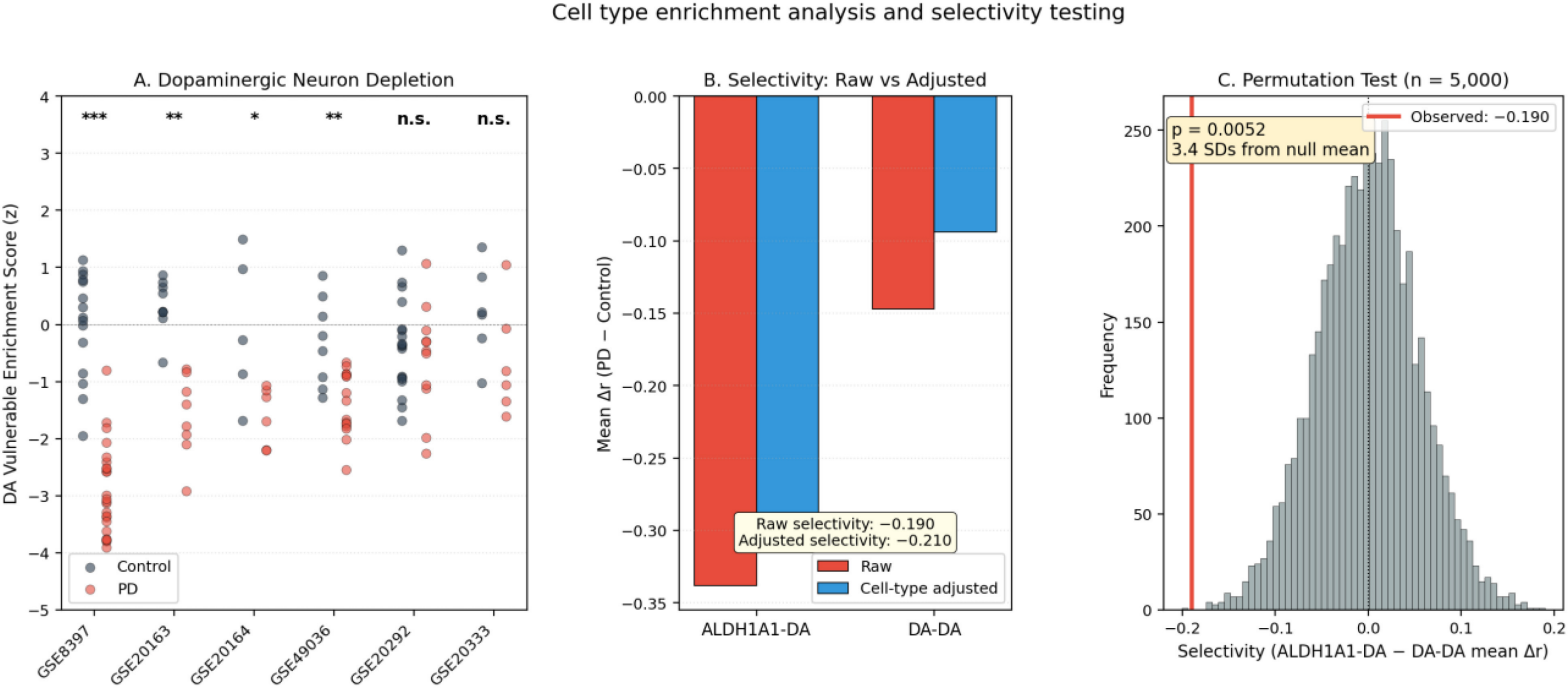
Cell type enrichment analysis and selectivity testing. **(A)** ALDH1A1-positive vulnerable dopaminergic neuron enrichment scores (z-scored mean expression of Kamath et al., 2022 markers, excluding target genes) in control and PD samples across six datasets. Significance indicators (Welch’s *t*-test): ***p < 0.001, **p < 0.01, *p < 0.05, ns, not significant. **(B)** Raw vs. cell-type-adjusted selectivity for ALDH1A1-DA and DA-DA categories; after cell-type adjustment, DA-DA attenuation shrinks substantially while ALDH1A1-DA attenuation is largely preserved. **(C)** Permutation null distribution (*n* = 5,000) for the selectivity statistic; the observed selectivity (–0.190) is marked in red and corresponds to *p* = 0.0052 (3.4 SDs from the null mean).

Marker gene-based enrichment scoring (Figure 4A) using Kamath et al. (2022) single-nucleus RNA-seq cell type markers confirmed significant depletion of ALDH1A1-positive vulnerable dopaminergic neurons in PD samples across 4 of 6 datasets: GSE8397 (Cohen’s *d* = 2.94, *p* < 0.001), GSE20163 (*d* = 1.46, *p* = 0.009), GSE20164 (*d* = 1.86, *p* = 0.014), and GSE49036 (*d* = 1.35, *p* = 0.006). Two datasets (GSE20292 and GSE20333) showed non-significant dopaminergic neuron depletion (*p* > 0.14), which may reflect sample heterogeneity, platform differences, or limitations of the enrichment scoring approach. The failure to detect significant dopaminergic neuron depletion in these two datasets—despite this being among the most robust pathological findings in PD—suggests that the marker gene-based enrichment approach has limited sensitivity in some dataset/platform combinations and should be interpreted as providing approximate rather than definitive composition estimates. NNLS-derived proportion estimates confirmed these patterns independently. Detailed enrichment analysis results, including per-dataset enrichment scores, NNLS proportions, and raw versus adjusted correlations, are reported in Supplementary Table 5.

**FIGURE 5:**
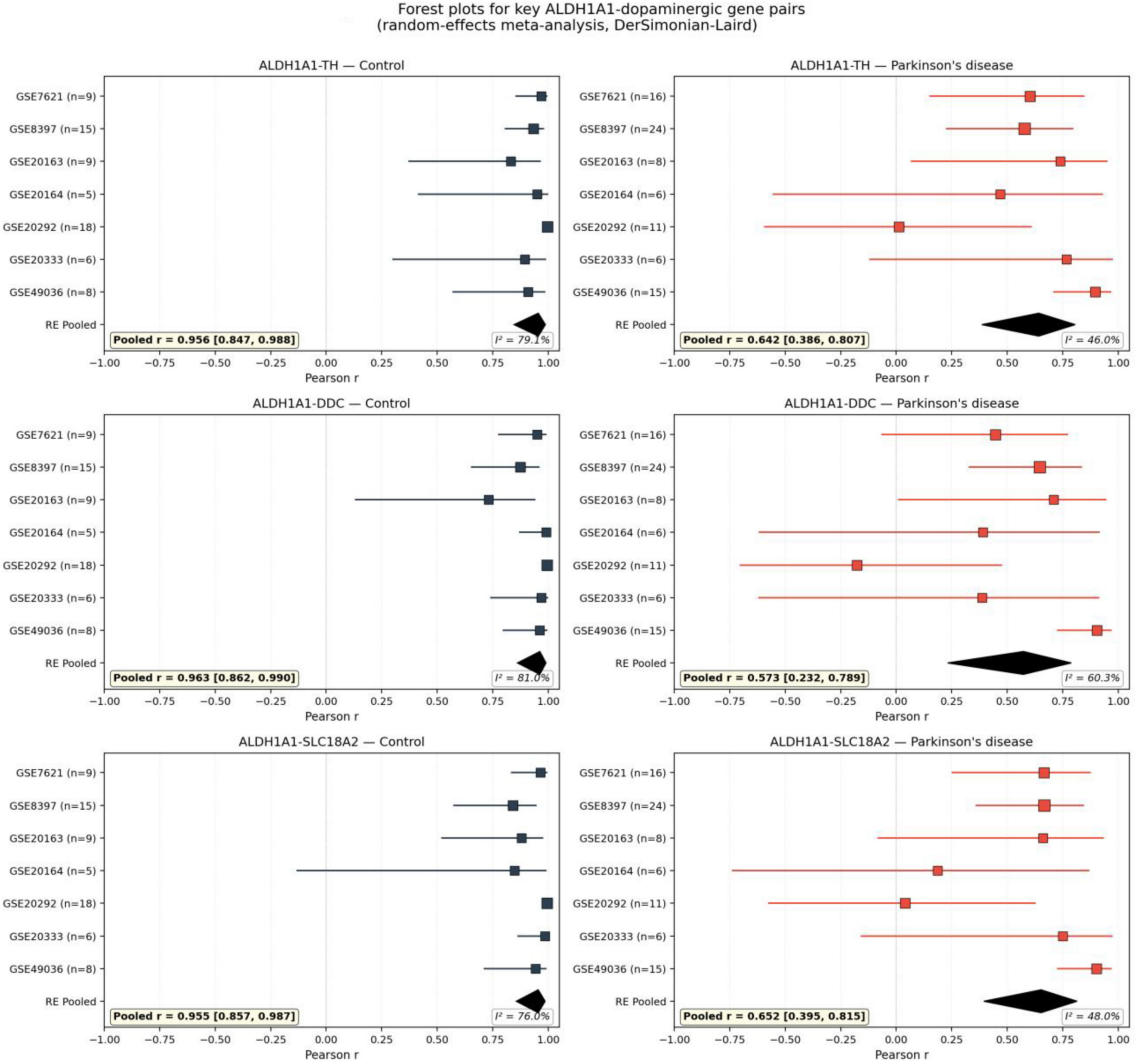
Forest plots for three key ALDH1A1-dopaminergic gene pairs (ALDH1A1-TH, ALDH1A1-DDC, ALDH1A1-SLC18A2) in control (blue) and Parkinson’s disease (red) conditions. Each panel shows individual dataset Pearson r (square markers, size proportional to n) with 95% Fisher’s z confidence intervals; the black diamond at bottom represents the random-effects pooled estimate (DerSimonian-Laird). Inset boxes report the pooled r with 95% CI and the I^2^ heterogeneity statistic.

After adjusting correlations for estimated ALDH1A1-positive dopaminergic neuron enrichment via partial correlation, the selectivity of ALDH1A1 attenuation was preserved and in fact increased. Adjusted ALDH1A1-dopamine Δr was −0.304, while adjusted dopamine-dopamine Δr was reduced from −0.147 to −0.094, yielding an adjusted selectivity difference of −0.210 (Figure 4B). The permutation null distribution for the selectivity statistic is shown in Figure 4C. Forest plots for the three primary ALDH1A1-dopaminergic pairs (ALDH1A1-TH, ALDH1A1-DDC, ALDH1A1-SLC18A2) are provided in Figure 5. Permutation testing of the adjusted selectivity was not performed because the partial correlation adjustment introduces additional estimation uncertainty that the current permutation framework does not model; the significant raw permutation result (*p* = 0.0052) together with the increased selectivity after adjustment support the robustness of the finding.

The increase in selectivity after adjustment is informative: dopamine-dopamine pair attenuation was partially attributable to cell composition changes (reduced from Δr = −0.147 to −0.094 after adjustment), whereas ALDH1A1 pair attenuation was less affected by adjustment (from Δr = −0.338 to −0.304), suggesting that factors beyond cell composition contribute to ALDH1A1 correlation loss.

### 3.8 Negative control gene pairs

Housekeeping gene pairs showed variable correlation changes across datasets (per-dataset values reported in Supplementary Table 6). The mean housekeeping Δr across the six enrichment analysis datasets was −0.106, though this average masks substantial dataset-level heterogeneity: GSE20292 exhibited extreme housekeeping instability (GAPDH-ACTB: Δr = −0.872; UBC-PPIA: Δr = −1.231), while other datasets showed modest changes (e.g., GSE8397 GAPDH-ACTB: Δr =+0.016). The comparable magnitude of housekeeping (−0.106) and dopamine-dopamine (−0.143) correlation attenuation is itself informative. Housekeeping genes, which share no specific biological pathway and are expressed across all cell types, provide an empirical estimate of non-specific tissue-level correlation instability arising from RNA degradation, postmortem interval effects, and sample processing heterogeneity. That dopamine-dopamine pair attenuation is of similar magnitude suggests that much of the dopamine-dopamine correlation change reflects these non-specific effects rather than pathway-specific disruption—consistent with the partial correlation analysis showing that DA-DA attenuation was substantially reduced after cell type adjustment (from −0.147 to −0.094). The ALDH1A1-dopamine Δr (−0.336) exceeds both comparators by approximately threefold, and this excess persists after cell type adjustment (adjusted Δr = −0.304), suggesting a disruption beyond what global tissue instability or proportional cell loss alone would predict. The relevant comparison is thus not ALDH1A1 attenuation versus zero, but ALDH1A1 attenuation versus the empirical tissue-level baseline established by both housekeeping and dopamine-dopamine pair changes.

### 3.9 Sensitivity analysis

Leave-one-out sensitivity analysis confirmed the robustness of the primary findings. For all ALDH1A1-dopamine pairs, sequentially excluding each dataset yielded qualitatively consistent results (Supplementary Table 3).

To address the question of whether the selectivity finding would replicate across independently handled datasets, we treated the full seven-dataset analysis as discovery and the four datasets with independently validated dopaminergic neuron depletion (GSE8397, GSE20163, GSE20164, GSE49036; see section “3.7 Cell type enrichment analysis”) as a technical-validation subset. This stratification was defined on the basis of enrichment-analysis quality, not on the basis of the selectivity outcome itself; the enrichment analysis is independent of the correlation-selectivity computation. Restriction to the validation subset yielded the strongest selectivity observed in any subset analysis (selectivity = −0.231; Welch’s *t* = −2.421, *p* = 0.021; Mann-Whitney *p* = 0.007; Cohen’s *d* = −0.787). This strengthening of the effect in the higher-quality subset, rather than attenuation, indicates that the discovery-phase finding is not an artifact of including lower-quality datasets in the pooled analysis; it replicates and amplifies in datasets where cell-type composition changes are independently verified. Selectivity sensitivity analysis across dataset subsets and gene pair definitions is reported in Supplementary Table 7. We explicitly acknowledge that this constitutes technical rather than biological independence—the datasets are drawn from the same design space (human postmortem SN microarray)—and note that replication in RNA-seq datasets and in independent cohorts not included in the present meta-analysis remains an important future direction.

When GSE20292—which exhibited extreme housekeeping gene correlation instability (GAPDH-ACTB: Δr = −0.872) and non-significant dopaminergic neuron depletion—was excluded, selectivity was maintained (selectivity = −0.118; Mann-Whitney *p* = 0.094), confirming that the primary findings are not driven by this potentially compromised dataset.

When SLC6A3 was excluded from the ALDH1A1-dopamine category (retaining only TH, DDC, and SLC18A2), the mean ALDH1A1-dopamine Δr increased in magnitude to −0.385, strengthening the selectivity finding. SLC6A3 was retained in the primary analysis because it was selected *a priori* as a core dopaminergic pathway gene, and *post hoc* exclusion risks data dredging. The heterogeneous behavior of SLC6A3 is discussed in section “4.2 Selective attenuation in Parkinson’s disease.”

## 4 Discussion

This systematic meta-analysis of gene expression correlations across seven independent human substantia nigra datasets demonstrates that ALDH1A1 shows consistently strong co-expression with dopaminergic pathway genes in neurologically normal tissue, and that these correlations are attenuated in Parkinson’s disease. The attenuation of ALDH1A1-dopamine correlations (mean Δr = −0.336) exceeds that of dopamine-dopamine correlations (mean Δr = −0.143), and this selectivity persists after adjusting for estimated cell type enrichment changes (adjusted selectivity: −0.210; permutation *p* = 0.0052). These findings are correlational and cannot distinguish among the multiple mechanisms that could produce this pattern, but they are consistent with a role for ALDH1A1-related processes in dopaminergic neuron vulnerability.

### 4.1 ALDH1A1-dopaminergic co-expression in healthy tissue

The strong correlations between ALDH1A1 and dopaminergic genes in healthy substantia nigra (mean *r* > 0.92 for TH, DDC, and SLC18A2) are consistent with several non-mutually exclusive explanations. First, ALDH1A1 and these dopaminergic genes are co-expressed within the same cell type—ventral tier SNpc dopaminergic neurons—and bulk tissue correlations may simply reflect this shared cellular origin. Second, they may share transcriptional regulatory mechanisms; both ALDH1A1 and dopaminergic genes are downstream targets of transcription factors including Nurr1 (NR4A2) and Pitx3, which are critical for dopaminergic neuron identity (Jacobs et al., 2009). Third, there may be functional coordination between retinoic acid signaling and dopamine metabolism, as retinoic acid has been shown to regulate TH expression and dopamine synthesis in experimental systems. However, the current analysis examined only ALDH1A1, not downstream RA signaling components (receptors, binding proteins, degradation enzymes). Whether broader RA pathway gene coordination is disrupted in PD cannot be determined from these data. The RA hypothesis remains one of several plausible interpretations; others include ALDH1A1’s role in dopamine aldehyde detoxification and its function as a subtype-specific marker independent of enzymatic activity. These possibilities are not distinguishable from the current correlational data.

We emphasize that the present study does not claim to identify a novel biological mechanism. Rather, it provides the first systematic cross-dataset quantification of ALDH1A1-dopaminergic co-expression changes in PD, demonstrating that the selective vulnerability of ALDH1A1-positive neurons—previously established at the single-cell level (Kamath et al., 2022)—produces a measurable and consistent transcriptomic signature in bulk tissue that is only partially attributable to cell composition changes.

### 4.2 Selective attenuation in Parkinson’s disease

The observation that ALDH1A1-dopamine correlations are attenuated more than dopamine-dopamine correlations is the central finding of this study. Multiple mechanisms could produce this pattern, and it is important to consider each. Preferential loss of ALDH1A1-positive neurons would reduce the ALDH1A1 signal in bulk tissue, attenuating correlations between ALDH1A1 and any co-expressed gene. This mechanism is well-established: Kamath et al. (2022) demonstrated that ALDH1A1-enriched dopaminergic neurons selectively degenerate in PD. Transcriptional dysregulation within surviving neurons—where ALDH1A1 expression declines independently of cell death—could also contribute. Galter et al. (2006) and others have reported ALDH1A1 downregulation that appears to exceed what cell loss alone would predict, though this is difficult to disentangle in bulk tissue. Post-transcriptional changes, epigenetic modifications, and altered protein turnover represent additional possibilities. The current data cannot determine the relative contributions of these mechanisms.

The ALDH1A1-SLC6A3 pair showed the smallest attenuation (Δr = −0.189), which is notable because the control correlation was also the weakest (mean *r* = 0.715). This may reflect the known heterogeneity of SLC6A3 expression within dopaminergic neuron subpopulations, as DAT expression varies with terminal field projection targets and is not strictly coupled to ALDH1A1 status. When SLC6A3 was excluded from the ALDH1A1-dopamine category, the mean Δr increased in magnitude to approximately −0.385, suggesting that SLC6A3 may follow a partially distinct regulatory program. SLC6A3 was retained in the primary analysis as an *a priori*-selected core dopaminergic gene to avoid *post hoc* exclusion bias.

### 4.3 Cell type enrichment considerations

A central concern in interpreting bulk tissue correlation changes is the potential confound of altered cell type composition. PD substantia nigra contains fewer dopaminergic neurons and relatively more glial cells, which could affect correlation structure independently of any transcriptional changes within surviving neurons. Our marker gene-based enrichment scoring using Kamath et al. (2022) single-nucleus RNA-seq markers—with all target genes excluded from cell type signatures to prevent circularity—confirmed significant dopaminergic neuron depletion in 4 of 6 datasets with large effect sizes (Cohen’s *d* = 1.35–2.94).

Importantly, the selectivity of ALDH1A1 attenuation was not diminished by cell type adjustment; rather, it increased from −0.190 (raw) to −0.210 (adjusted). The mechanism underlying this increase is informative: after partialing out dopaminergic neuron enrichment, dopamine-dopamine pair attenuation was reduced from Δr = −0.147 to −0.094, indicating that cell composition changes partially account for dopamine-dopamine pair attenuation. In contrast, ALDH1A1 pair attenuation was less affected by adjustment (from −0.338 to −0.304), suggesting that cell composition changes alone do not account for ALDH1A1 correlation loss. The direction of this asymmetry—dopamine-dopamine attenuation partially explained by cell loss, ALDH1A1 attenuation largely independent of it—is consistent across datasets and argues against regression artifact as an explanation, since noisy covariate adjustment would be expected to add random directional noise rather than systematically increase selectivity.

Important caveats apply to this analysis. Our approach used marker gene-based enrichment scoring and NNLS regression rather than full reference-based deconvolution through the CIBERSORTx web portal with complete single-cell transcriptome profiles. While the NNLS algorithm is mathematically equivalent to CIBERSORTx’s core method, the marker gene approach provides approximate rather than precise proportion estimates. Two of six datasets (GSE20292 and GSE20333) showed non-significant dopaminergic neuron depletion, which may reflect sample quality issues or limitations of the enrichment scoring approach. While partial correlations controlled for dopaminergic neuron subtypes, residual confounding from other cell type shifts (increased microglia, astrogliosis) was not fully addressed to maintain statistical power given the modest per-dataset sample sizes. Restricting analysis to the four datasets with validated deconvolution yielded stronger selectivity (−0.256, *p* = 0.006), suggesting that the enrichment approach is adequate when marker gene mapping is sufficient. Formal deconvolution using complete single-cell reference panels and simultaneous adjustment for multiple cell types represents an important direction for future validation.

### 4.4 SNCA and broader network considerations

SNCA-containing gene pairs showed the largest correlation attenuation (mean Δr = −0.548), exceeding both ALDH1A1-dopamine (−0.336) and dopamine-dopamine (−0.143) pairs. The uniformly large SNCA attenuation across all partner genes is consistent with α-synuclein’s central role in PD pathogenesis, potentially reflecting disease-specific pathological processes including synuclein aggregation, impaired proteostasis, and associated transcriptional disruption. The SNCA findings were not the primary focus of this study but warrant dedicated investigation. The triangulation analysis in section “3.5.1 Triangulation against non-specific transcriptional collapse” uses the SNCA pattern as a disease-global baseline against which the partner-specific ALDH1A1 selectivity can be evaluated.

### 4.5 Limitations

Several important limitations warrant consideration. First, and most fundamentally, correlation does not establish causation. The observed correlation changes could reflect any combination of cell death, transcriptional dysregulation, post-transcriptional regulation, technical artifacts, or other factors. The designation of changes as “attenuation” is purely descriptive and does not imply a specific biological mechanism.

Second, postmortem tissue reflects end-stage disease, and the interpretation of bulk SN expression data is subject to a survivor-selection bias that warrants explicit consideration. Because microglial phagocytosis clears degenerated neurons relatively rapidly (Chitnis and Weiner, 2017; see also Venegas and Heneka, 2017), tissue obtained at autopsy preferentially samples surviving neurons—those that withstood the neurodegenerative process, plus smaller cohorts still undergoing active degeneration at time of death. The vulnerable ALDH1A1-enriched population that selectively degenerates in PD (Kamath et al., 2022) is therefore underrepresented in postmortem tissue relative to its abundance in the pre-symptomatic SN. This has an important implication for the interpretation of ALDH1A1 attenuation: the signal we detect is measured after microglial clearance has already removed a substantial fraction of the cells that would otherwise have contributed to the correlation structure. The persistence of detectable ALDH1A1-dopamine correlation attenuation even in this survivor-enriched tissue is therefore consistent with any combination of three mechanisms: (i) active degeneration continuing at time of death, contributing cells in intermediate states; (ii) chronic stress-related transcriptional reorganization within surviving neurons, in which ALDH1A1 coupling to dopaminergic machinery is disrupted without cell death; and (iii) residual depletion of the ALDH1A1-positive subpopulation even within the survivor-enriched pool, as reported at the single-cell level by Kamath et al. (2022). These mechanisms are not mutually exclusive, and the present bulk data cannot adjudicate among them. Longitudinal studies in prodromal PD and single-cell transcriptomic analyses of paired affected/resistant subpopulations would be needed to dissect these contributions. We note that this survivor bias, rather than negating the observed signal, reframes it: detectable ALDH1A1 attenuation in surviving tissue suggests that the transcriptional coupling disruption extends beyond the cells that have already been lost to neurodegeneration.

Third, all datasets used microarray platforms, which have lower dynamic range and different technical characteristics than RNA-seq. Validation using RNA-seq datasets would strengthen confidence in the observed patterns.

Fourth, although individual dataset sample sizes were modest (*n* = 11–39), the combined meta-analytic sample (*n* = 156) and the consistency across all seven datasets mitigate concerns about statistical power. Nevertheless, the small per-group sample sizes in some datasets (as low as *n* = 5) limit the precision of individual correlation estimates. The I^2^ = 0% observed for ALDH1A1-dopamine pairs in PD, while suggesting low heterogeneity, should be interpreted cautiously in this context, as low heterogeneity statistics can arise when within-study variance is large relative to between-study variation (Borenstein et al., 2009). The consistency of direction and approximate magnitude across all seven datasets provides qualitative support independent of formal heterogeneity assessment.

Fifth, the cell type enrichment analysis used marker gene-based scoring rather than formal methods with complete single-cell reference profiles. While the zero-circularity design and the permutation-based significance testing address key methodological concerns, more precise deconvolution could alter the quantitative estimates.

Sixth, several potential confounders could not be addressed in this analysis, including medication effects (most PD patients were on L-DOPA therapy), postmortem interval, RNA quality, and demographic factors. These variables were inconsistently reported across datasets. The permutation test, which shuffles disease labels within datasets, implicitly assumes exchangeability of samples conditional on dataset membership. Because disease status is confounded with these unmeasured variables, the permutation null distribution may underestimate true null variance, potentially inflating significance. However, this limitation applies to all permutation-based inference on observational data and does not favor an alternative statistical approach; parametric tests on non-independent gene pair observations would introduce different and arguably more severe violations. The consistency of the selectivity pattern across all seven datasets—each with different sample characteristics, platforms, and investigators—provides qualitative support that is independent of any single statistical test.

Seventh, several datasets in this meta-analysis (GSE20163, GSE20164, GSE20292) were generated by overlapping investigator groups. Comparison of GEO sample accession identifiers confirmed no overlapping GSM IDs between GSE20163 and GSE20164 or between GSE20292 and GSE20333, indicating that these datasets represent distinct sample submissions. However, because different GSM accessions can correspond to the same biological specimen profiled in different submissions, partial sample overlap at the tissue level cannot be definitively excluded. If present, this would reduce the effective independence of observations and could inflate apparent cross-dataset consistency. Leave-one-out sensitivity analysis demonstrated that no single dataset was indispensable to the primary findings, partially mitigating this concern, but future analyses should attempt to identify and exclude overlapping samples using donor-level identifiers where available.

Eighth, the negative control analysis revealed that housekeeping gene pairs show variable and sometimes substantial correlation changes in PD tissue, with marked dataset-level heterogeneity. This underscores the caution needed in interpreting any bulk tissue correlation change as biologically specific.

### 4.6 Future directions

Several research directions emerge from these correlational observations. Single-cell RNA-seq analysis of PD and control substantia nigra would enable within-cell-type correlation analysis, definitively resolving whether ALDH1A1-dopamine co-expression changes reflect cell loss or transcriptional dysregulation within surviving neurons. Experimental perturbation of ALDH1A1 expression or retinoic acid levels in dopaminergic neuron cultures or animal models would test the functional hypothesis that RA pathway disruption affects dopaminergic gene co-regulation. Longitudinal studies examining ALDH1A1 pathway biomarkers in prodromal PD cohorts could assess whether correlation changes precede clinical diagnosis. Replication in emerging RNA-seq SN case-control datasets and in independent cohorts not included in the present meta-analysis would strengthen external validity. Dedicated investigation of SNCA co-expression patterns, which showed the largest and most uniform attenuation in this analysis, is warranted as a distinct research question.

## 5 Conclusion

Meta-analysis of gene expression correlations across seven independent human substantia nigra datasets reveals that ALDH1A1 shows consistently strong co-expression with dopaminergic pathway genes in healthy tissue, and that these correlations are attenuated in Parkinson’s disease with cross-dataset consistency. The attenuation is greater for ALDH1A1-containing pairs than for dopamine-dopamine pairs, and this selectivity persists after marker gene-based cell type enrichment adjustment (permutation *p* = 0.0052). While these correlational findings cannot establish causation or definitively distinguish cell loss from transcriptional dysregulation, they provide a quantitative framework for understanding ALDH1A1-dopaminergic relationships in PD and are consistent with the hypothesis that retinoic acid pathway disruption contributes to dopaminergic vulnerability. Mechanistic studies are warranted to determine whether ALDH1A1 downregulation is a cause, consequence, or epiphenomenon of neurodegeneration.

## Data Availability

Publicly available datasets were analyzed in this study. The GEO datasets can be found at the following URLs: https://www.ncbi.nlm.nih.gov/geo/query/acc.cgi?acc=GSE7621; https://www.ncbi.nlm.nih.gov/geo/query/acc.cgi?acc=GSE8397; https://www.ncbi.nlm.nih.gov/geo/query/acc.cgi?acc=GSE20163; https://www.ncbi.nlm.nih.gov/geo/query/acc.cgi?acc=GSE20164; https://www.ncbi.nlm.nih.gov/geo/query/acc.cgi?acc=GSE20292; https://www.ncbi.nlm.nih.gov/geo/query/acc.cgi?acc=GSE20333; and https://www.ncbi.nlm.nih.gov/geo/query/acc.cgi?acc=GSE49036. The complete analysis code for this meta-analysis is publicly available at https://github.com/nwharbert8-ui/ALDH1A1-PD-meta-analysis-Repo.

## Supplementary material

Supplementary Table 1 Quality assessment of included datasets.

Supplementary Table 2 Individual dataset correlation values for all gene pairs in control and PD.

Supplementary Table 3 Leave-one-out sensitivity analysis results.

Supplementary Table 4 Cell type marker gene lists used for enrichment scoring (Kamath et al., 2022), demonstrating exclusion of all target genes.

Supplementary Table 5 Detailed enrichment analysis results by dataset, including enrichment scores, NNLS proportions, and raw vs. adjusted correlations.

Supplementary Table 6 Negative control housekeeping gene pair correlations by dataset.

Supplementary Table 7 Sensitivity analysis of selectivity across dataset subsets and gene pair definitions.
