# Supplementary material for: ALDH1A1-dopaminergic gene co-expression in human substantia nigra: meta-analysis of disease-associated correlation changes across seven independent Parkinson’s disease datasets
Source: Front Aging Neurosci. 2026 May 19;18:1806505. doi: 10.3389/fnagi.2026.1806505 (PMC13226206; doi:10.3389/fnagi.2026.1806505)
Supplement: Supplementary file 1 [file Image_1.pdf]

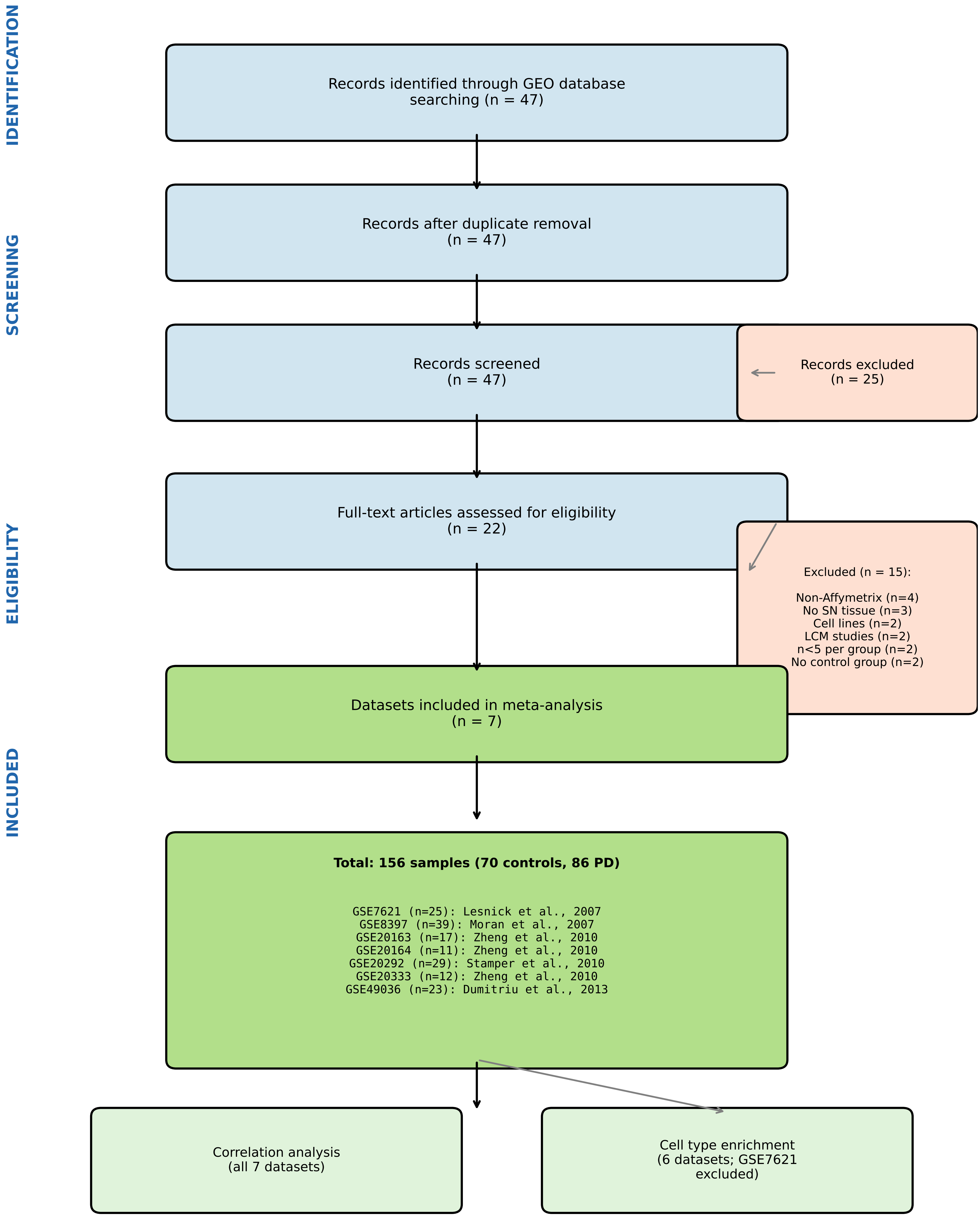

Supplementary Figure S1. PRISMA flow diagram for dataset identification and selection.
