# Supplementary material for: ALDH1A1-dopaminergic gene co-expression in human substantia nigra: meta-analysis of disease-associated correlation changes across seven independent Parkinson’s disease datasets
Source: Front Aging Neurosci. 2026 May 19;18:1806505. doi: 10.3389/fnagi.2026.1806505 (PMC13226206; doi:10.3389/fnagi.2026.1806505)
Supplement: Supplementary file 2 [file Data_Sheet_8.docx]

**Supplementary Table S8.** Z-test comparison of pooled control versus PD correlation coefficients for all gene pairs.

Pooled correlation coefficients were estimated via DerSimonian–Laird random-effects meta-analysis of Fisher’s z-transformed correlations across seven independent substantia nigra microarray datasets (k = 7; n = 156 total; 70 controls, 86 PD). Z-tests compare pooled control and PD correlations using the formula: z = (z_ctrl − z_PD) / √(SE²_ctrl + SE²_PD), where z_ctrl and z_PD are the pooled Fisher’s z estimates and SE values are the corresponding random-effects standard errors. Two-sided p-values < 0.05 were considered statistically significant. Δr = Pooled r(PD) − Pooled r(Control).

| **Gene Pair** | **Category** | **k** | **Pooled r (Ctrl)** | **Pooled r (PD)** | **Δr** | **z** | **p-value** | **Sig.** |
| --- | --- | --- | --- | --- | --- | --- | --- | --- |
| ALDH1A1-TH | ALDH1A1-DA | 7 | 0.955 | 0.720 | **-0.235** | **3.45** | **<0.001** | **Yes** |
| ALDH1A1-SLC18A2 | ALDH1A1-DA | 7 | 0.958 | 0.725 | **-0.233** | **3.06** | **0.002** | **Yes** |
| ALDH1A1-DDC | ALDH1A1-DA | 7 | 0.962 | 0.671 | **-0.291** | **2.92** | **0.004** | **Yes** |
| ALDH1A1-SLC6A3 | ALDH1A1-DA | 7 | 0.871 | 0.680 | -0.190 | 0.90 | 0.370 | No |
| DDC-TH | DA-DA | 7 | 0.925 | 0.842 | -0.082 | 1.33 | 0.183 | No |
| SLC18A2-TH | DA-DA | 7 | 0.919 | 0.844 | -0.076 | 1.23 | 0.218 | No |
| DDC-SLC18A2 | DA-DA | 7 | 0.970 | 0.937 | -0.032 | 0.82 | 0.411 | No |
| SLC18A2-SLC6A3 | DA-DA | 7 | 0.909 | 0.860 | -0.048 | 0.53 | 0.598 | No |
| SLC6A3-TH | DA-DA | 7 | 0.866 | 0.873 | +0.006 | -0.05 | 0.957 | No |
| DDC-SLC6A3 | DA-DA | 7 | 0.852 | 0.849 | -0.003 | 0.02 | 0.982 | No |
| SLC18A2-SNCA | SNCA | 7 | 0.948 | 0.535 | **-0.413** | **3.36** | **<0.001** | **Yes** |
| SNCA-TH | SNCA | 7 | 0.938 | 0.535 | **-0.403** | **3.35** | **<0.001** | **Yes** |
| DDC-SNCA | SNCA | 7 | 0.937 | 0.494 | **-0.443** | **3.13** | **0.002** | **Yes** |
| ALDH1A1-SNCA | SNCA | 7 | 0.957 | 0.635 | **-0.322** | **3.08** | **0.002** | **Yes** |
| SLC6A3-SNCA | SNCA | 7 | 0.822 | 0.413 | -0.409 | 1.61 | 0.108 | No |

**Gene pair categories:** ALDH1A1-DA (yellow shading) = ALDH1A1 paired with dopaminergic pathway genes (TH, DDC, SLC18A2, SLC6A3); DA-DA (white) = pairwise combinations among dopaminergic pathway genes; SNCA (green shading) = α-synuclein paired with any target gene. Bold values indicate statistically significant comparisons (p < 0.05). Within each category, pairs are sorted by ascending p-value.

**Key finding:** Three of four ALDH1A1-dopamine pairs show significant attenuation in PD (all p ≤ 0.004), while none of the six DA-DA pairs reach significance (all p > 0.18). The non-significant ALDH1A1-SLC6A3 pair (p = 0.370) is consistent with the heterogeneous behavior of SLC6A3 across dopaminergic neuron subpopulations (see main text, Section 4.2). Among SNCA-containing pairs, four of five show significant attenuation (all p ≤ 0.002), with only SLC6A3-SNCA non-significant (p = 0.108), again reflecting SLC6A3 heterogeneity.
